# A Nationally Representative Survey Assessing Restorative Sleep in US Adults

**DOI:** 10.3389/frsle.2022.935228

**Published:** 2022-07-21

**Authors:** Rebecca Robbins, Stuart F. Quan, Daniel J. Buysse, Matthew D. Weaver, Matthew P. Walker, Christopher L. Drake, Kristen Monten, Laura K. Barger, Shantha M. W. Rajaratnam, Thomas Roth, Charles A. Czeisler

**Affiliations:** ^1^Division of Sleep and Circadian Disorders, Departments of Medicine and Neurology, Brigham and Women's Hospital, Boston, MA, United States; ^2^Division of Sleep Medicine, Harvard Medical School, Boston, MA, United States; ^3^Department of Psychiatry, University of Pittsburgh School of Medicine, Pittsburgh, PA, United States; ^4^Department of Psychology, Center for Human Sleep Science, University of California, Berkeley, Berkeley, CA, United States; ^5^Sleep Disorders and Research Center, Henry Ford Hospital, Detroit, MI, United States; ^6^Middlebury College, Middlebury, VT, United States; ^7^School of Psychological Sciences, Turner Institute for Brain and Mental Health, Monash University, Melbourne, VIC, Australia; ^8^Institute for Breathing and Sleep, Austin Health, Heidelberg, VIC, Australia

**Keywords:** restorative sleep, sleep health, sleep, national sample, survey

## Abstract

Restorative sleep is a commonly used term but a poorly defined construct. Few studies have assessed restorative sleep in nationally representative samples. We convened a panel of 7 expert physicians and researchers to evaluate and enhance available measures of restorative sleep. We then developed the revised Restorative Sleep Questionnaire (REST-Q), which comprises 9 items assessing feelings resulting from the prior sleep episode, each with 5-point Likert response scales. Finally, we assessed the prevalence of high, somewhat, and low REST-Q scores in a nationally representative sample of US adults (*n* = 1,055) and examined the relationship of REST-Q scores with other sleep and demographic characteristics. Pairwise correlations were performed between the REST-Q scores and other self-reported sleep measures. Weighted logistic regression analyses were conducted to compare scores on the REST-Q with demographic variables. The prevalence of higher REST-Q scores (4 or 5 on the Likert scale) was 28.1% in the nationally representative sample. REST-Q scores positively correlated with sleep quality (*r* = 0.61) and sleep duration (*r* = 0.32), and negatively correlated with both difficulty falling asleep (*r* = −0.40) and falling back asleep after waking (*r* = −0.41). Higher restorative sleep scores (indicating more feelings of restoration upon waking) were more common among those who were: ≥60 years of age (OR = 4.20, 95% CI: 1.92–9.17); widowed (OR = 2.35, 95% CI: 1.01–5.42), and retired (OR = 2.02, 95% CI: 1.30–3.14). Higher restorative sleep scores were less frequent among those who were not working “other” (e.g., a person performing household duties, OR = 0.36, 95% CI: 0.10–1.00) and living in a household with two or more persons (OR = 0.51, 95% CI: 0.29–0.87). Our findings suggest that the REST-Q may be useful for assessing restorative sleep.

## Introduction

There has been an explosion in the availability and uptake of consumer technologies for tracking sleep duration and other quantitative sleep metrics. According to a nationally representative survey, 25% of US adults have used a smartphone or device to track their sleep duration (Robbins et al., 2018). While interest in tracking sleep among the population suggests increased interest and awareness about sleep, quantitative assessments do not capture a holistic, qualitative (i.e., self-reported) evaluation of sleep (Buysse, 2014). For instance, while few differences in quantitative measures of sleep are observed between insomnia patients and healthy controls, striking differences are seen in the qualitative evaluations of sleep (Orff et al., 2007; Corsi-Cabrera et al., 2016). Several initiatives have been made to capture qualitative evaluations, such as perceptions of restoration or quality after waking from sleep, using questionnaires (Buysse, 2014; Drake et al., 2014; Matsumoto et al., 2017; Nakajima et al., 2018; Balanzá-Martínez et al., 2021). Despite the importance of qualitative assessments of sleep, such as feelings of restoration from sleep, little research has comprehensively evaluated qualitative evaluations of sleep in a representative sample of the US adult population.

While restorative sleep is not often measured, *non*-restorative sleep has been widely measured (Ohayon and Partinen, 2002; Ohayon, 2005; Ohayon and Sagales, 2010; Roth et al., 2010). Non-restorative sleep is defined as the subjective evaluation of sleep as being unrefreshing that is not accounted for by lack of sleep/sleep opportunity (Stone et al., 2008). Historically, non-restorative sleep has been a distinct component of several definitions of insomnia disorder, including the 4th edition of the Diagnostic and Statistical Manual of Mental Disorders (DSM-4), but not the more recent 5th edition (DSM-5) (American Psychiatric Association, 2013). In part due to the characterization of non-restorative sleep as an insomnia symptom, many questionnaires for assessing insomnia in clinic settings include single items assessing non-restorative sleep (Grandner and Kripke, 2004; Nakajima et al., 2018; Garefelt et al., 2020; Balanzá-Martínez et al., 2021). Studies relying upon such single items (e.g., “Do you ever wake up with a feeling of exhaustion and fatigue”), many of which feature simple yes/no response options (Wakasugi et al., 2014; Nakajima et al., 2018), have examined the prevalence of non-restorative sleep in diverse samples, including general adult populations, insomnia patients, and patients with a variety of other chronic illnesses, and yielded a wide range of prevalences from 8% to 42% (Ohayon and Partinen, 2002; Phillips and Mannino, 2005; Ohayon and Bader, 2010; Zhang et al., 2012; Matsumoto et al., 2017). Another limitation of these studies is that the question wording used to assess non-restorative sleep also ranged widely. For instance, several studies have asked participants to report the times they woke and “felt unrefreshed,” while other times they were asked if they felt “restored,” or if they woke and felt “unrested” without distinguishing from sleep deprivation effects by excluding assessments after nights with inadequate sleep. Drake et al. developed a validated measure for assessing non-restorative sleep that was rigorously tested among insomnia patients and healthy controls, performing well on reliability and validity tests (Drake et al., 2014). In the study conducted by Drake and colleagues, correlation analyses revealed that restorative sleep responses were weakly correlated with sleep duration (*r* = 0.32) and there was only a moderate correlation with sleep quality (ranging *r* = 0.46–0.59 depending on the scale assessing sleep quality). Although originally designed to measure non-restorative sleep, the questions on the Drake et al. survey solicit responses to questions that relate to positive evaluations of sleep, such as feeling “ready to start the day” and “energetic” after waking, which capture assessments of the restorative properties of sleep as rated by the sleeper (as opposed to the non-restorative properties). Nevertheless, the work from Drake and colleagues suggests that restorative sleep may be an important, independent construct to measure. In addition, the tool developed by Drake and colleagues to measure restorative sleep has not been widely used. Moreover, we lack a conceptual definition of restorative sleep. We recruited a panel of expert sleep specialists to address this gap and develop a conceptual definition of restorative sleep.

Our first aim was to reach consensus with a panel of expert sleep specialists on a definition of restorative sleep, then to review, critique, and enhance the measure of restorative sleep offered by Drake and colleagues (Drake et al., 2014), so as to arrive at a reliable, easy-to-use tool for assessing restorative sleep (REST-Q). Finally, we assessed the prevalence of low, somewhat, and high REST-Q scores in a nationally representative sample of US adults and examined the relationship of REST-Q scores with other sleep and demographic characteristics.

## Materials and Methods

### Overview

First, we aimed to reach consensus with a panel of expert sleep specialists on a definition of restorative sleep. Second, we reviewed and critiqued available measures of restorative sleep, Finally, we explored the prevalence of low, somewhat, and high REST-Q scores in a nationally representative sample of adults in the US and examined the relationship of REST-Q scores with other sleep and demographic characteristics.

### Literature Review

In order to identify available measures of restorative sleep, we conducted a series of literature searches. The literature searches were conducted using the term “restorative sleep” in Medline and Psych Info. Searches resulted in 366 articles. The articles were screened to identify studies that actually *measured* restorative or non-restorative sleep. After the screening was complete, 58 articles were eligible, including 10 that measured restorative sleep and 48 that measured non-restorative sleep. The eligible articles resulted in a pool of 32 different measures of either restorative or non-restorative sleep, which were shared with the experts prior to the expert panel discussion and presented by the first author to the experts during the panel discussion.

### Expert Panel to Define Restorative Sleep and Develop the Restorative Sleep Questionnaire

In accordance with the RAND Delphi procedure (Dalkey and Helmer, 1963), we recruited expert sleep medicine physicians and scientists (*n* = 7) to reach a definition of restorative sleep and review, critique, and enhance the available measures of restorative sleep.

Experts were selected based upon demonstrated expertise, as measured by the number of peer-reviewed publications, in the following domains: survey design and psychometrics in sleep and circadian rhythms; sleep medicine and circadian rhythms disorders; and both restorative and non-restorative sleep research. Consistent with the RAND Delphi Procedure, the experts convened for a series of linked steps. The first step included a focus group where experts were prompted to develop a definition of restorative sleep and critique available measures (Drake et al., 2014). The first step resulted in a document with a preliminary definition of restorative sleep and a list of proposed questions for assessing restorative sleep. The second step included final editorial changes to the definition and measures. In the third step, the definition and draft questions were sent to the experts who were asked to provide responses on 9-point scales of appropriateness to the definition of restorative sleep, the utility and appropriateness of each measure of restorative sleep on the proposed questionnaire, and the proposed method for scoring. Appropriateness was rated on scales from 1 (not at all appropriate) to 9 (extremely appropriate).

The experts agreed that the questionnaire developed by Drake et al. (2014) was the most thorough measure, but proposed minor changes to wording and scoring. Specifically, experts proposed the instructions be changed from “For each question, circle the number that best indicates how you feel” to “For each of the following items, please tell me to what degree you feel each of the below when you woke up today, compared to before you went to sleep. Last night's sleep left me feeling” followed by a series of 9 words or phrases (e.g., “…tired?,” “…sleepy?,” “…in a good mood?,” and “…rested?”). Drake and colleagues proposed a method for scoring the responses to their questionnaire but did not propose categories to distinguish between those who were low vs. high on restorative sleep. We propose these modifications in the Restorative Sleep Questionnaire (REST-Q), a 9-item questionnaire assessing aspects of restorative sleep. Finally, we propose a simple formula for scoring the REST-Q which results in three categories of restorative sleep (low, somewhat, and high), based on the average response participants make to the questionnaire.

### Nationally Representative Panel Participants & Procedures

Surveys were administered to *AmeriSpeak*, a probability-based panel managed by the National Opinion Research Center (NORC) at the University of Chicago. Amerispeak is designed to be representative of the US household population. Randomly selected US households are sampled using area probability and address-based sampling. The sampled households are then contacted by US mail, telephone, and field interview (face to face). Participants in the AmeriSpeak panel are then invited to join subsequent panels annually by web or telephone. Participants provide written informed consent during enrollment in the panel. Participants for the present study were a stratified random sample of panelists drawn from the AmeriSpeak panel. Sample stratification was employed to assure representativeness with respect to age, gender, race/ethnicity, and education. To ensure representativeness of the sample, our team compared the resultant sample to data from the US Census Bureau (data.census.gov). The study sample is representative of the US adult population with respect to age, gender, education, and race/ethnicity of US adults (see the Supplementary Material A for statistics from the US Census Bureau: data.census.gov).

Participants were able to complete surveys in English or Spanish. Eligible participants included adults (18 years of age or older) residing in a US household. The current survey was sent to 5,259 participants from the AmeriSpeak panel in September 2021. The survey took ~15 min to complete. One thousand and fifty-fifth participants completed the survey for a 20.06% completion rate. Among the respondents, 7% of interviews were conducted by phone and 93% online (34% on a desktop, 57% on a smartphone, and 2% on a tablet).

### Survey Measures

On the nationally representative survey, we assessed demographic, sleep, and REST-Q variables. Demographic characteristics measured in the present study included gender, age, race/ethnicity, education, marital status, employment status, household income, living in an urban (vs. non-urban) area, home internet access, home ownership, and number of persons living in the household.

Sleep variables measured included sleep duration, sleep quality, self-reported insomnia, and sleep difficulties. Sleep duration before work or school days was measured by asking individuals “During the past month, on average, how many hours of actual sleep did you obtain before a typical work or school day?” and before free days by asking “…before a typical ‘free’ day, that is a non-work, non-school day?,” consistent with previous research (Robbins et al., 2021). A measure of average weekly sleep duration was created by computing a weighted average of sleep durations reported for work/school and free nights, assuming the reported work/school night sleep duration was maintained for five nights in a typical week and the reported free night sleep duration was maintained for 2 nights in a typical week. Sleep quality was measured by asking participants “During the past month, how would you rate your sleep quality overall” from 1 (very poor), 2 (poor), 3 (fair), 4 (good), and 5 (very good), consistent with the PROMIS sleep questionnaire (Full et al., 2019). Participants were asked if they have ever received an insomnia diagnosis (yes or no). Finally, the frequency of sleep disturbances was measured by asking participants “During the past month how often did it take you more than 30 min to fall asleep at night?” and “…how often did you have trouble falling aback asleep on nights after waking?” Sleep disturbance responses were collected on scales from 1 (every night), 2 (most nights), 3 (some nights), 4 (rarely), and 5 (never), then reverse coded so that higher values indicate more disturbance.

The REST-Q asked participants “For each of the following items, please tell me to what degree you feel each of the below when you woke up today, compared to before you went to sleep. Last night's sleep left me feeling…” with 9 different words or phrases to following: Restorative Sleep Question 1 (RSQ1): “…tired;” RSQ2: “…sleepy;” RSQ3: “…in a good mood;” RSQ4: “…rested;” RSQ5: “…refreshed;” RSQ6: “…ready to start the day;” RSQ7: “…energetic;” RSQ8: “…mentally alert;” and RSQ9: “…grouchy.” Responses were captured on a scale from 1 (not at all) to 2 (a little bit), 3 (somewhat), 4 (very much), and 5 (completely). Responses to “…tired,” “…sleepy,” and “…grouchy” were reverse coded.

Responses to the 9 REST-Q questions were averaged then transformed to a 100-point scale, consistent with Drake et al. (2014) (see the Formula below). Then, we proposed that the transformed value be categorized into one of three overall scores based on the corresponding value from the original 5-point Likert scale. Specifically, a score of 50 corresponded to an average response of “not at all” or “a little bit” to the restorative sleep questions and would be categorized as a “low” REST-Q score. Scores ranging from 50 to 74.99 corresponded to an average response of “somewhat” to the restorative sleep questions and would be categorized as a “somewhat” REST-Q score. Finally, scores of 75 and above corresponded to an average response of “very much” or “completely” to the restorative sleep questions and would be categorized as a “high” REST-Q score.


$$\left[\left(\frac{RSQ1+RSQ2+RSQ3+RSQ4+RSQ5+RSQ6+RSQ7+RSQ8+RSQ9}{9}\right)-1\right]\times 25$$

### Statistical Analysis

Representativeness of the US population was achieved by using weighted proportions with the svy command in Stata statistical software (Version 16; StataCorp, College Station, TX). Internal consistency of the REST-Q items was determined using Cronbach's alpha. Demographic characteristics of the sample stratified by REST-Q score (low, somewhat, high) were compared using Pearson χ^2^ statistics. Descriptive statistics were captured for each of the 9 items on the REST-Q and plotted to determine the frequency distribution of responses. Pairwise correlations were performed between the REST-Q transformed (0–100) values and sleep variables (sleep duration on weekdays, sleep duration on free days, sleep quality, difficulty falling asleep, and nighttime awakenings). Mean scores on the sleep variables (sleep duration on weekdays, sleep duration on free days, sleep quality, difficulty falling asleep, and nighttime awakenings) by REST-Q score (low, somewhat, high) were tested using ANOVA. The prevalence in this nationally representative panel of REST-Q scores (low, somewhat, and high) were tabulated. Finally, weighted logistic regression analyses were conducted to compare those with a high score on the REST-Q (compared to low or somewhat) by each demographic variable. Two-sided hypothesis tests were used with p <0.05 considered to be the threshold for statistical significance.

## Results

### Results From the Expert Panel

**Figure 1:**
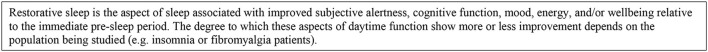
Definition of restorative sleep from the panel of expert sleep medicine physicians and researchers.

The definition of restorative sleep (Figure 1) developed through the Delphi procedure received a mean appropriateness rating of 7.6/9 (S.D. = 1.6) from the 7 experts. The REST-Q and the associated scoring procedure developed through the Delphi procedure received a mean appropriateness rating of 8.3/9 (S.D. = 0.82) from the experts.

In the nationally representative survey to assess responses to the REST-Q, participants (*n* = 1,055) were 48% male and 52% female participants and average age was 49.4 years (S.D. = 17.5 years). Restorative sleep scores as measured by the REST-Q varied by marital status (*p* < 0.037), employment status (*p* < 0.001), urban vs. rural area (*p* < 0.05), and number of people living in a household (*p* < 0.01, Table 1).

**Table 1:** Demographic characteristics of the sample by Restorative Sleep Questionnaire (REST-Q) score.

		**REST-Q Score**													
		**Low**			**Somewhat**			**High**			**Total**				
		***n*** **= 340**			***n*** **= 435**			***n*** **= 280**			***n*** **= 1,055**			**Chi**	***P-***
		***N^W^***	***N^A^***	***%^W^***	***N^W^***	***N^A^***	***%^W^***	***N^W^***	***N^A^***	***%^W^***	***N^W^***	***N^A^***	***%^W^***	**square**	**value**
Gender	Male	156	159	31	223	243	44	131	149	26	510	551	48	2.8	0.070
	Female	189	181	35	190	192	35	166	131	30	545	504	52		
Age	18–29	94	68	45	80	56	38	35	21	17	209	145	20	**8.0**	**0.000**
	30–44	111	128	40	120	142	43	48	52	17	279	322	26		
	45–59	85	76	35	95	85	39	66	58	27	246	219	23		
	60+	54	68	17	119	152	37	148	149	46	321	369	30		
Race/Ethnicity	White, non-Hispanic	223	220	34	243	272	37	195	198	30	661	690	63	0.7	0.681
	Black, non-Hispanic	37	39	29	55	61	43	34	34	27	126	134	12		
	Other, non-Hispanic	3	5	55	1	3	13	2	3	31	6	11	1		
	Hispanic	55	52	31	81	65	46	40	32	23	176	149	17		
	More than one	11	17	50	7	14	29	5	4	21	23	35	2		
	Asian, non-Hispanic	16	7	26	28	20	43	20	9	31	64	36	6		
Education	Less than HS	23	10	25	38	12	40	33	10	35	95	32	9	1.9	0.092
	HS graduate or equivalent	132	74	44	102	66	34	68	42	22	303	182	29		
	Some college/associates	97	154	34	119	189	42	70	109	24	286	452	27		
	Bachelor's degree	56	59	26	95	107	43	68	64	31	220	230	21		
	Grad/professional degree	36	43	24	59	61	39	58	55	38	152	159	14		
Marital status	Married	150	150	29	212	232	41	151	158	29	514	540	49	**2.2**	**0.037**
	Widowed	6	7	17	12	15	33	17	14	49	35	36	3		
	Divorced	30	33	28	35	37	32	43	33	40	107	103	10		
	Separated	16	13	31	22	22	41	15	15	28	52	50	5		
	Never married	112	108	39	118	105	41	58	42	20	288	255	27		
	Living with partner	30	29	51	16	24	27	13	18	22	59	71	6		
Employment	Working (paid employee)	176	196	33	203	223	38	151	145	29	530	564	50	**3.1**	**0.001**
	Working (self)	19	24	34	23	32	42	13	15	24	55	71	5		
	Not working (e.g., layoff)	10	8	69	2	5	15	2	3	16	15	16	1		
	Not working (looking)	45	27	49	36	32	39	11	7	12	92	66	9		
	Not working (retired)	30	29	15	81	89	40	90	86	45	201	204	19		
	Not working (disabled)	38	31	46	25	25	30	19	13	23	82	69	8		
	Not working (other)	26	25	33	44	29	55	10	11	13	80	65	8		
Household income	<$30,000	104	103	35	123	106	42	68	53	23	296	262	28	0.8	0.572
	$30,000 to under $60,000	90	91	33	101	121	38	78	79	29	269	291	25		
	$60,000 to under $100,000	90	85	34	97	109	36	79	85	30	267	279	25		
	$100,000 or more	60	61	27	93	99	42	63	70.8	28	224	223	21		
Urban vs. Rural	Non-urban area	76	63	42	56	65	31	47	48	26	180	176	17	**3.8**	**0.024**
	Urban area	269	277	31	358	370	41	249	232	28	876	879	83		
Internet access	No home access	49	43	36	53	55	39	35	33	25	137	131	13	0.3	0.737
	Home internet access	296	297	32	361	380	39	262	247	29	918	924	87		
Home ownership	Owned	226	188	32	253	257	36	218	192	31	697	637	66	2.3	0.065
	Rented for cash	111	142	34	144	159	44	75	84	23	329	385	31		
	Occupied without payment	8	10	28	17	19	59	4	4	13	29	33	3		
Household size	I live by myself	40	52	23	71	80	40	67	65	37	177	197	17	**2.8**	**0.009**
	2 persons	103	107	29	125	145	35	128	131	36	356	383	34		
	3 persons	58	57	40	55	65	37	34	32	23	147	154	14		
	4 persons	52	54	35	69	70	46	28	29	19	149	153	14		
	5 persons	33	35	38	47	34	53	8	8	9	88	77	8		
	+6 persons	59	35	43	48	41	35	31	15	23	138	91	13		

*Bold indicates significance at the p < 0.05 level*.

W *Represents weighted estimates*.

A *Represents unweighted/actual estimates*.

**Figure 2:**
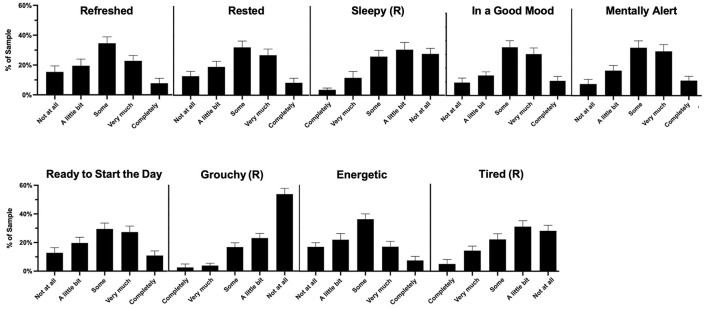
Descriptive statistics summarizing responses to the 9 questions which comprise the Restorative Sleep Questionnaire (REST-Q). Each question featured the stem “For each of the following items, please tell me to what degree you felt each of the below when you woke up today, compared to before you went to sleep. Last night's sleep left me feeling…”.

The items on the REST-Q demonstrated internal consistency with a Cronbach's alpha of 0.92 and inter-item covariance of 0.65. The responses to the individual REST-Q questions were normally distributed except for sleepy, grouchy and tired which were right skewed (Figure 2).

### Results From the Nationally Representative Panel

**Figure 3:**
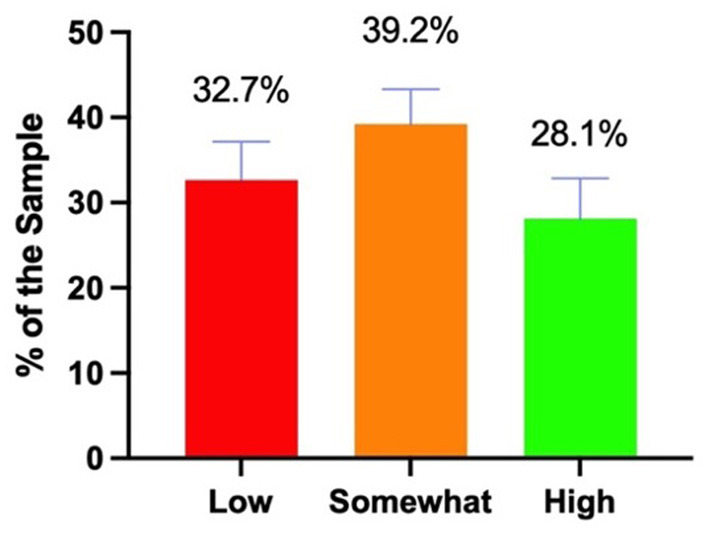
Prevalence of low, somewhat, and high REST-Q scores in a nationally representative sample of US adults.

The weighted prevalence of high restorative sleep based on the REST-Q was 28.1% in this nationally representative panel (Figure 3).

**Figure 4:**
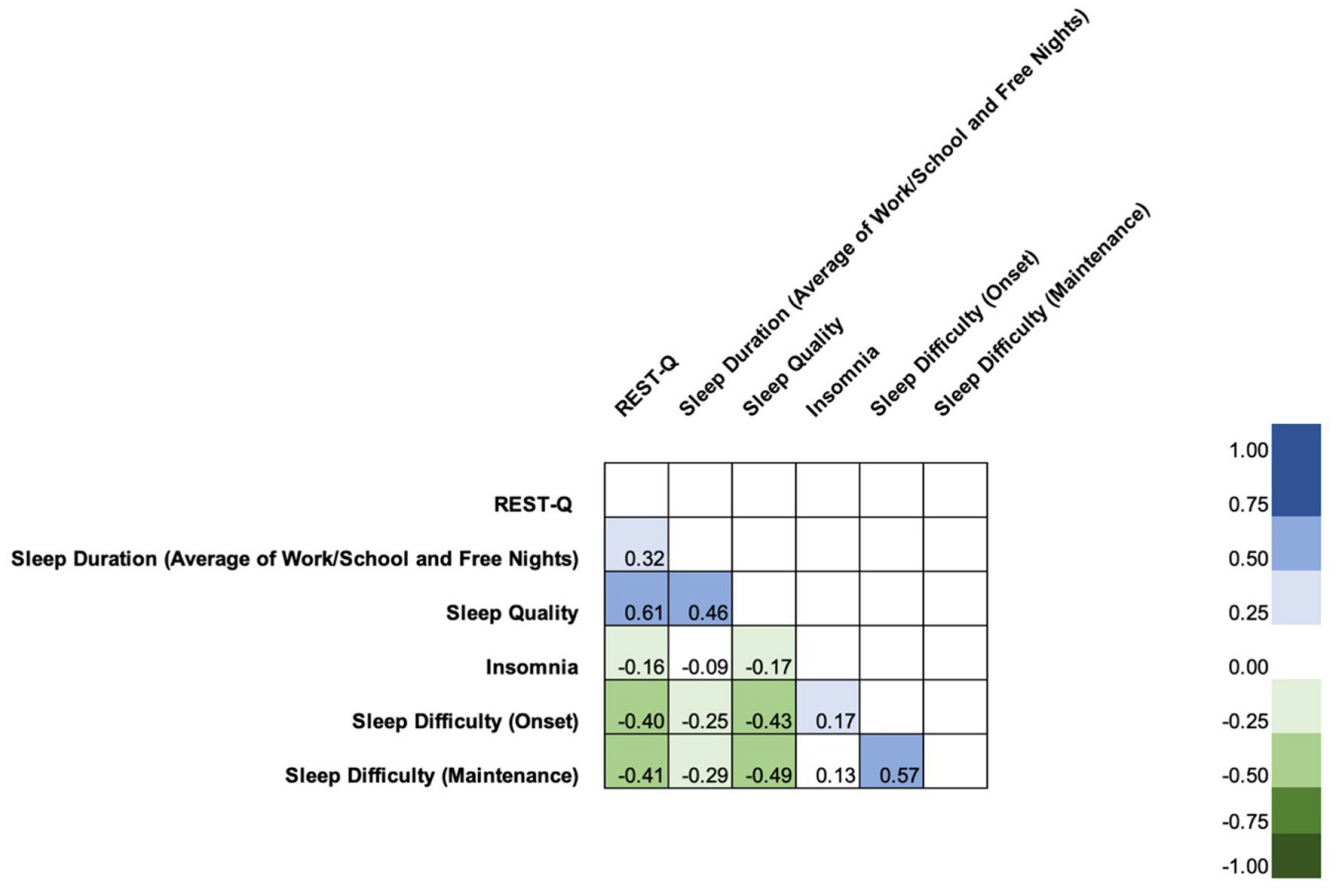
Pairwise correlation matrix of REST-Q responses (on the 100-point scale) and sleep duration, sleep quality, insomnia, and sleep difficulties (onset and maintenance). The REST-Q scores used in the correlation analyses are the 100-point scores that have not yet been scored to the “low,” “somewhat,” and “high” categories. Color indicates the magnitude and direction of the correlation. Bright green indicates a strong, negative correlation and light green indicates a weak, negative correlation. Bright blue indicates a strong, positive correlation and light blue indicates a weak, positive correlation. The sleep duration variable displayed is the weighted weekly average sleep duration, with 5/7^th^ weight assigned to the reported sleep duration on work/school nights and 2/7^th^ weight assigned to the reported sleep duration on free nights.

REST-Q scores were positively correlated with the weighted average of weekly sleep duration (*r* = 0.32, *p* < 0.001) and sleep quality (*r* = 0.61, *p* < 0.001). REST-Q scores were negatively correlated with self-reported insomnia diagnoses (*r* = −0.16, *p* < 0.001) and sleep difficulties (onset: *r* = −0.40, *p* < 0.001; maintenance: *r* = −0.41, *p* < 0.001, Figure 4).

**Figure 5:**
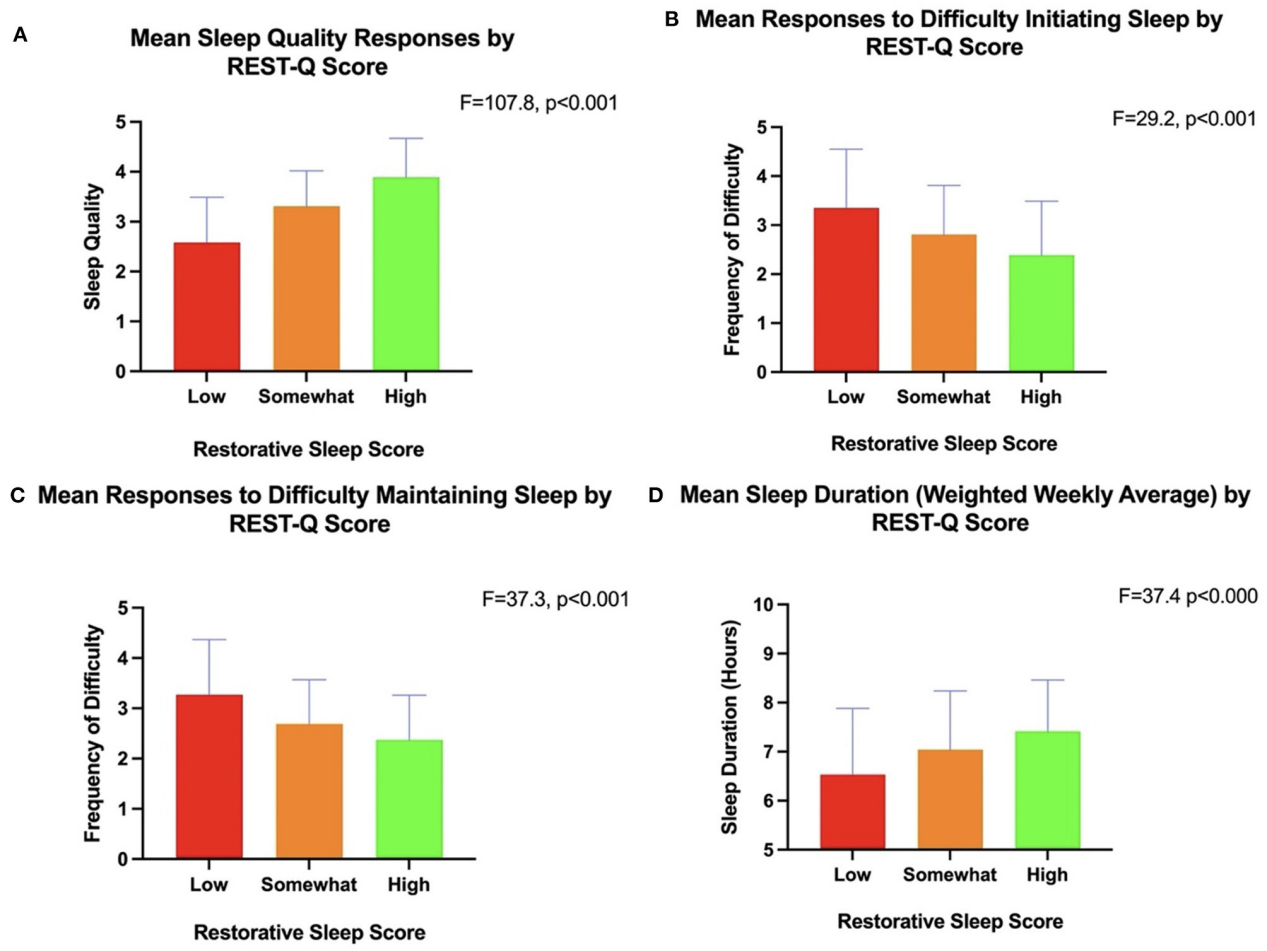
Mean responses regarding sleep quality **(A)**, difficulty initiating sleep **(B)**, difficulty maintaining sleep **(C)**, weighted average of week/school and free night sleep duration **(D)** by REST-Q scores (low, somewhat, high). The sleep duration variable used in the ANOVA displayed in **(D)** is the weighted weekly average sleep duration, with 5/7th weight assigned to the reported sleep duration on work/school nights and 2/7th weight assigned to the reported sleep duration on free nights.

Sleep quality (*F* = 107.8, *p* < 0.001), sleep duration (*F* = 37.4, *p* < 0.001), difficulty initiating sleep (*F* = 29.2, *p* < 0.001), and difficulty maintaining sleep (*F* = 37.3, *p* < 0.001) all varied by REST-Q score (Figure 5).

Weighted logistic regression analyses indicated that the following demographic characteristics were associated with *greater* odds of restorative sleep as scored by the REST-Q: age 60 years or older compared to age 18–25 (OR = 4.20, 95% CI: 1.92–9.17); being widowed compared to being married (OR = 2.35, 95% CI: 1.01–5.42); and being retired compared to working as a paid employee (OR = 2.02, 95% CI: 1.30–3.14). Conversely, the following factors were associated with *reduced* odds of higher restorative sleep as scored by the REST-Q: not working (other, e.g., a person performing household duties) compared to working as a paid employee (OR = 0.36, 95% CI: 0.15–0.89); renting a residence (OR = 0.65, 95% CI: 0.43–0.97) or occupying a residence without payment (OR = 0.034, 95% CI: 0.32–0.10) as compared to owning a residence; and residing with 3 other persons (OR = 0.51, 95% CI :0.29–0.87), 4 other persons (OR = 0.39, 95% CI: 0.24–0.64), or 5 other persons (OR = 0.17, 95% CI:0.07–0.40) compared to living alone (see Supplementary Information).

## Discussion

Qualitative evaluation is an important feature of sleep health (Buysse, 2014), yet the vast majority of high quality nationally representative data collected among US adults has assessed quantitative aspects, such as sleep duration, which preclude a holistic understanding of sleep in the population. Moreover, the practice of tracking quantitative sleep measures, such as nightly sleep duration and even sleep staging, has become common among US adults, yet few of these technologies afford users the opportunity to provide qualitative or subjective ratings of their sleep, such as restorative sleep. Our study convened an expert panel to develop a definition of restorative sleep, propose a measure of restorative sleep, then capture nationally representative data regarding the response rates of this measure in comparison to other metrics of sleep quality among US adults.

Our study addresses conceptual ambiguity which has persisted in the sleep field with regards to restorative sleep. For instance, our literature search returned more than 350 articles from a keyword search for “restorative sleep,” but only 48 *measured* non-restorative or restorative sleep. Upon further exploration, we discovered that “restorative” was often used in studies as a synonym for sufficient sleep duration or satisfaction with sleep, such as high ratings on sleep quality. With a panel of survey design, psychometrics, sleep, and circadian rhythms experts, we found support for a definition that positions restorative sleep as an aspect of sleep that is indicative of the restoration of positive daytime characteristics, such as improved mood, energy, and wellbeing. It is possible that such a definition, which makes clear the connection between sleep and daytime outcomes, may aid in increasing sleep's importance among the general population. Furthermore, the definition of restorative sleep provided here is aligned with the call for more attention to quantifying optimal sleep health, as opposed to poor sleep health (Buysse, 2014). The expert panel also provided strong support for the REST-Q as a tool for assessing restorative sleep, providing high ratings for the measure on scales of appropriateness.

In a nationally representative panel, we explored the prevalence of REST-Q scores and found that high restorative sleep scores were observed in 28% of US adults. Whereas, previous nationally representative data has found markers of sleep health, such as sufficient sleep duration, in two thirds of US adults (Liu, 2016), our findings indicate that less than one third of US adults received high scores for restorative sleep. We also examined demographic characteristics with respect to scores on the REST-Q, finding that higher scores were more likely among those age 60 and above, those who were widowed, and those who reported being retired. These findings are consistent with previous research in a large convenience sample of Japanese adults, which found that reports of *non*-restorative sleep declined with age (Wakasugi et al., 2014), suggesting that older individuals, perhaps due to less stress associated with raising children or fewer professional obligations among those who are retired, increases the likelihood of restorative sleep. This finding is somewhat contradictory to other studies, which demonstrate increased reports of sleep difficulties among older adults as compared to younger adults (Ohayon, 2002). In addition, we found that being widowed was associated with higher odds of restorative sleep compared to being married. This finding may be reflect that sleeping with a partner can be disruptive, either due to different sleep/wake times maintained by either partner or due to one (or both) individuals snoring (Pevernagie et al., 2010; Blumen et al., 2012). It was surprising that we did not detect a gender difference in our data. Previous research has shown that rates of sleep difficulties, such as sleep dissatisfaction, are higher in females than in males (Ohayon, 2002). Overall, our findings contrast those from the insomnia literature, which have shown that the disorder is more common among women than men and more common among older as compared to younger adults (Ohayon, 2002). In contradistinction, our study did not find a statistically significant difference in REST-Q scores by gender and found a statistically significant difference between ages, such that older adults were more likely to have higher REST-Q scores than younger adults. Taken together, our findings, demonstrating higher odds of restorative sleep among older adults and widowed individuals as well as higher markers of sleep health in younger adults, suggest that restorative sleep may not simply the converse of non-restorative sleep, or other insomnia symptoms, but a distinct feature of sleep entirely. Future research is needed to examine restorative sleep as measured by the REST-Q and other biological or physiological measures to explore REST-Q responses and markers of physical and emotional health and wellbeing.

We also observed that higher scores on the REST-Q were positively associated with better sleep quality and longer sleep duration on work and free day and inversely associated with sleep difficulties, including difficulty falling asleep and waking up from sleep without being able to fall back asleep. While there were significant associations between the REST-Q and sleep quality, sleep duration, insomnia, sleep onset and sleep maintenance, correlation analyses were only weak to moderate. Our study is consistent with previous research that suggests that non-restorative sleep has independent associations with chronic health conditions after controlling for insomnia symptoms (Zhang et al., 2012), indicating that non-restorative sleep is a construct that is unique from other sleep complaints. By extension, it is possible that restorative sleep is similarly distinct, and not the mere converse of insomnia symptoms. This suggests that while there is overlap, the REST-Q is capturing a conceptual aspect of sleep distinct from other evaluations of sleep, which we believe reflects restorative sleep. Nevertheless, future research is needed to explore further how the general population views the feeling of restoration upon waking and how that experience is similar to or distinct from other appraisals of sleep, such as reports of sleep quality.

We propose that our findings demonstrate that the REST-Q is a reliable tool for assessing restorative sleep, with high convergent validity and internal consistency. Also, our research is the first nationally representative study to evaluate the performance of a measure designed to assess restorative sleep among a nationally representative sample of US adults. Despite these strengths, our work has several limitations. First, our study did not have access to chronic disease diagnoses from the participants. Previous research has found that non-restorative sleep is common among certain conditions, such as depression (Müller et al., 2017) and fibromyalgia (Azad et al., 2000), but no research to our knowledge has examined *restorative sleep* and chronic conditions. It is important to note that the present study did not measure sleep disorders other than insomnia, such as obstructive sleep apnea. Future research may examine comorbid conditions and/or sleep disorders and restorative sleep as measured by the REST-Q. Second, we were not able to schedule the time of day of survey administration. Future research may explore the issue of timing of delivery of the REST-Q. For instance, researchers may administer the REST-Q at several post-sleep intervals (e.g., 2, then 4, then 6 h after waking) to explore how feelings of restoration change over the day, and perhaps identify the optimal time for administration of the REST-Q tool. Third, we did not measure chronotype, which refers to the timing of the internal circadian clock relative to light-dark cycles in one's external environment (Aschoff, 1965). Research has demonstrated evening chronotypes underperform in the morning hours compared to their morning chronotype counterparts (Ritchie et al., 2017). Future research may explore how time of day and chronotype matter for REST-Q responses. Fourth, the present study did not administer the REST-Q at different points in time, which precluded determination of test-retest reliability of the assessment tool. Future researchers may evaluate the REST-Q in a prospective study to examine how restorative sleep evolves over time and relates to daytime behaviors in addition to sleep. Future research may also undertake additional psychometric analyses with the REST-Q, such as qualitative research with patients to get input on the face validity of the REST-Q. Finally, it is a limitation in the present study that the scored REST-Q responses are categorized as “low,” “somewhat,” or “high” based on the corresponding scale value (e.g., a score of 50 corresponded to an average response of “somewhat” to the questions on the REST-Q). Future research may test the REST-Q categories against additional criteria, such as actigraphy-derived sleep efficiency.

In summary, our study convened a panel of expert sleep medicine specialists and sleep scientists to develop a consensus definition and derive a new measure of restorative sleep. We administered the REST-Q to a nationally representative sample, finding fewer than one third of US adults reported restorative sleep as assessed by this new measure. We identified demographic predictors of restorative sleep as measured by the REST-Q, namely age, marital status, employment status, household type, and household size as significant predictors of restorative sleep. Taken together, these findings suggest that restorative sleep may be an important metric to consider when assessing sleep health in population studies.

## Data Availability Statement

The raw data supporting the conclusions of this article will be made available by the authors, without undue reservation.

## Ethics Statement

The studies involving human participants were reviewed and approved by NORC (University of Chicago). Written informed consent was provided by the participants.

## Author Contributions

RR, SFQ, MDW, LKB, and CAC contributed to the conceptualization and the design the work. RR prepared the manuscript. All authors contributed to the acquisition, analysis, interpretation of the data, conceptualized the manuscript, and approved the submitted version.

## Funding

This work was supported by a grant from the Bryte Foundation. LKB, MDW, CAC, and RR are supported in part by NIOSH (R01OH011773) and NIH (R56HL151637). RR is supported by the NIH (K01HL150339).

## Conflict of Interest

RR has received consulting fees from Rituals Cosmetics BV, Savoir Beds Ltd, With Deep Inc, Sleep Cycle AB, Oura Ring Ltd, Denihan Hospitality Group, and the Skimm. MDW has received salary and/or institutional support from Delta Airlines, the National Sleep Foundation, Puget Sound Pilots, and the University of Pittsburgh. MPW serves as a consultant for and has equity interest in the companies, Bryte, Shuni, Oura Ring, and StimScience. SFQ reports receiving consulting fees from Whispersom, Bryte Foundation, Best Doctors, DR Capital. SR has unpaid appointments at CRC for Alertness, Safety and Productivity, Australia and the Sleep Health Foundation. SR also is supported on grants from Vanda Pharmaceuticals, Philips Respironics, Cephalon, Rio Tinto, BHP Billiton, and Shell. SR also has received other support from Optalert, Compumedics, Teva Pharmaceuticals, and Circadian Therapeutics. CAC reports grants and contracts to BWH from Dayzz Live Well, Delta Airlines, Jazz Pharma, Puget Sound Pilots, Regeneron Pharmaceuticals/Sanofi; is/was paid consultant/speaker for Inselspital Bern, Institute of Digital Media and Child Development, Klarman Family Foundation, M. Davis and Co, National Council for Mental Wellbeing, National Sleep Foundation, Physician's Seal, SRS Foundation, State of Washington Board of Pilotage Commissioners, Tencent, Teva Pharma Australia, With Deep, and Vanda Pharmaceuticals, in which CAC holds an equity interest; received travel support from Aspen Brain Institute, Bloomage International Investment Group, Inc., Dr. Stanley Ho Medical Development Foundation, German National Academy of Sciences, Ludwig-Maximilians-Universität München, National Highway Transportation Safety Administration, National Safety Council, National Sleep Foundation, Salk Institute for Biological Studies/Fondation Ipsen, Society for Research on Biological Rhythms, Stanford Medical School Alumni Association, Tencent Holdings, Ltd, and Vanda Pharmaceuticals; receives research/education gifts through BWH from Arbor Pharmaceuticals, Avadel Pharmaceuticals, Bryte, Alexandra Drane, Cephalon, DR Capital Ltd, Eisai, Harmony Biosciences, Jazz Pharmaceuticals, Johnson & Johnson, Mary Ann & Stanley Snider *via* Combined Jewish Philanthropies, NeuroCare, Inc., Optum, Philips Respironics, Regeneron, Regional Home Care, ResMed, San Francisco Bar Pilots, Sanofi SA, Schneider, Simmons, Sleep Cycle. Sleep Number, Sysco, Teva Pharmaceuticals, Vanda Pharmaceuticals; is/was an expert witness in legal cases, including those involving Advanced Power Technologies, Aegis Chemical Solutions, Amtrak; Casper Sleep Inc, C&J Energy Services, Catapult Energy Services Group, Covenant Testing Technologies, Dallas Police Association, Enterprise Rent-A-Car, Espinal Trucking/Eagle Transport Group/Steel Warehouse Inc, FedEx, Greyhound, Pomerado Hospital/Palomar Health District, PAR Electrical Contractors, Product & Logistics Services LLC/Schlumberger Technology, Puckett EMS, Puget Sound Pilots, Union Pacific Railroad, UPS, and Vanda Pharmaceuticals; serves as the incumbent of an endowed professorship given to Harvard by Cephalon; and receives royalties from McGraw Hill and Philips Respironics for the Actiwatch-2 and Actiwatch Spectrum devices. CAC interests were reviewed and are managed by the Brigham and Women's Hospital and Mass General Brigham in accordance with their conflict-of-interest policies. The remaining authors declare that the research was conducted in the absence of any commercial or financial relationships that could be construed as a potential conflict of interest. The authors declare that this study received funding from The Bryte Foundation. The funder was not involved in the study design, collection, analysis, interpretation of data, the writing of this article or the decision to submit it for publication.

## Publisher's Note

All claims expressed in this article are solely those of the authors and do not necessarily represent those of their affiliated organizations, or those of the publisher, the editors and the reviewers. Any product that may be evaluated in this article, or claim that may be made by its manufacturer, is not guaranteed or endorsed by the publisher.
