# Supplementary material for: A Nationally Representative Survey Assessing Restorative Sleep in US Adults
Source: Front Sleep. 2022 Jul 21;1:935228. doi: 10.3389/frsle.2022.935228 (PMC9423762; doi:10.3389/frsle.2022.935228)
Supplement: Supplementary file 1 [file Table_1.PDF]

Supplementary Information A

Descriptive statistics of US adults according to the US Census Bureau.

Variable	Category	%
**Age**	18-29	20.50%
	30-44	25.20%
	45-59	24.50%
	60+	29.80%
**Gender**	Male	48.30%
	Female	51.70%
**Education**	Less than high school diploma	9.80%
	High school diploma or equivalent	28.20%
	Some college/ associates	27.70%
	Bachelor's degree or above	34.30%
**Race/Ethnicity**	White, non-Hispanic	62.80%
	Black, non-Hispanic	11.90%
	Hispanic	16.70%
	Asian, non-Hispanic	6.40%
	Other	2.20%
