# Supplementary material for: A Nationally Representative Survey Assessing Restorative Sleep in US Adults
Source: Front Sleep. 2022 Jul 21;1:935228. doi: 10.3389/frsle.2022.935228 (PMC9423762; doi:10.3389/frsle.2022.935228)
Supplement: Supplementary file 2 [file Table_2.docx]

**Supplementary Information C**

**Results from the weighted logistic regressions examining the relationships between demographic factors and restorative sleep as scored on the REST-Q (low, somewhat, high).**

|  |  | **REST-Q Score** | | | |
| --- | --- | --- | --- | --- | --- |
|  |  |  | ***95 % Confidence Interval*** | |  |
| **Variable** | **Category** | ***Odds Ratio*** | ***Lower*** | ***Upper*** | ***P-Value*** |
| **Gender** | Male | Reference | | | |
|  | Female | 1.27 | 0.88 | 1.83 | 0.192 |
| **Age** | 18-29 | Reference | | | |
|  | 30-44 | 1.02 | 0.43 | 2.43 | 0.959 |
|  | 45-59 | 1.79 | 0.82 | 3.91 | 0.140 |
|  | 60+ | **4.20** | **1.92** | **9.17** | **0.000** |
| **Race/Ethnicity** | White, non-Hispanic | Reference | | | |
|  | Black, non-Hispanic | 0.90 | 0.49 | 1.66 | 0.736 |
|  | Other, non-Hispanic | 1.08 | 0.25 | 4.72 | 0.916 |
|  | Hispanic | 0.71 | 0.42 | 1.22 | 0.216 |
|  | More than one | 0.64 | 0.15 | 2.73 | 0.547 |
|  | Asian, non-Hispanic | 1.09 | 0.37 | 3.21 | 0.877 |
| **Education** | Less than high school diploma | Reference | | | |
|  | High school diploma or equivalent | 0.54 | 0.20 | 1.43 | 0.213 |
|  | Some college/ associates | 0.60 | 0.25 | 1.45 | 0.255 |
|  | Bachelor's degree | 0.84 | 0.33 | 2.13 | 0.705 |
|  | Grad/professional degree | 1.13 | 0.45 | 2.81 | 0.800 |
| **Marital Status** | Married | Reference | | | |
|  | Widowed | **2.35** | **1.01** | **5.42** | **0.046** |
|  | Divorced | 1.58 | 0.91 | 2.74 | 0.102 |
|  | Separated | 0.93 | 0.37 | 2.36 | 0.886 |
|  | Never married | 0.60 | 0.33 | 1.09 | 0.092 |
|  | Living with partner | 0.68 | 0.31 | 1.50 | 0.335 |
| **Employment Status** | Working (paid employee) | Reference | | | |
|  | Working (self) | 0.80 | 0.36 | 1.79 | 0.584 |
|  | Not working (temporary layoff) | 0.48 | 0.10 | 2.29 | 0.354 |
|  | Not working (looking for work) | 0.34 | 0.08 | 1.46 | 0.147 |
|  | Not working (retired) | **2.02** | **1.30** | **3.14** | **0.002** |
|  | Not working (disabled) | 0.76 | 0.30 | 1.92 | 0.565 |
|  | Not working (other) | **0.36** | **0.15** | **0.89** | **0.027** |
| **Household Income** | Less than $30,000 | Reference | | | |
|  | $30,000 to under $60,000 | 1.36 | 0.74 | 2.49 | 0.317 |
|  | $60,000 to under $100,000 | 1.41 | 0.85 | 2.32 | 0.178 |
|  | $100,000 or more | 1.54 | 0.89 | 2.66 | 0.120 |
| **Urban versus Rural** | Non-Metro Area | Reference | | | |
|  | Metro Area | 1.11 | 0.73 | 1.68 | 0.639 |
| **Internet Access** | No home access | Reference | | | |
|  | Home access | 1.18 | 0.72 | 1.94 | 0.517 |
| **Home Ownership** | Owned | Reference | | | |
|  | Rented for cash | **0.65** | **0.43** | **0.97** | **0.034** |
|  | Occupied without payment | **0.32** | **0.10** | **1.00** | **0.049** |
| **Household Size** | I live by myself | Reference | | | |
|  | 2 persons | 0.94 | 0.56 | 1.57 | 0.814 |
|  | 3 persons | **0.51** | **0.29** | **0.87** | **0.014** |
|  | 4 persons | **0.39** | **0.24** | **0.64** | **0.000** |
|  | 5 persons | **0.17** | **0.07** | **0.40** | **0.000** |
|  | +6 persons | 0.49 | 0.19 | 1.26 | 0.139 |

*Notes.*

Bold indicates significance at the p<0.05 level.

The outcome variable was dichotomized, coded so that 1 represents a score of “high” on the REST-Q and 0 represents a score of “low” or “somewhat” on the REST-Q.
