# Supplementary material for: A Nationally Representative Survey Assessing Restorative Sleep in US Adults
Source: Front Sleep. 2022 Jul 21;1:935228. doi: 10.3389/frsle.2022.935228 (PMC9423762; doi:10.3389/frsle.2022.935228)
Supplement: Supplementary file 3 [file Presentation_1.PDF]

Supplemental Information B
The Restorative Sleep Questionnaire (REST-Q)
Questionnaire and Scoring Procedure

Instruction: For each of the following items, please tell me to what degree you felt each of the below when you woke up today, compared to before you went to sleep. Last night's sleep left me feeling…

	Not at all	*A little bit*	*Somewhat*	*Very much*	*Completely*
	1	*2*	*3*	*4*	*5*
RSQ 1. ...Tired (R)					
RSQ 2. ...Sleepy(R)					
RSQ 3. ...In a good mood					
RSQ 4. ...Rested					
RSQ 5. ...Refreshed					
RSQ 6. ...Ready to start the day					
RSQ 7. ...Energetic					
RSQ 8. ...Mentally alert					
RSQ 9. ...Grouchy (R)					

Scoring: A total score on the REST-Q is calculated using the below formula. Scores range from 0 to 100. Total scores are then categorized into three levels: responses from 0 to 49.99 are assigned a label of "low;" responses from 50 to 74.99 are assigned a label of "somewhat;" and responses from 75 to 100 are assigned a label of "high" on the REST-Q.

$$\left[\left(\frac{RSQ1 + RSQ2 + RSQ3 + RSQ4 + RSQ5 + RSQ6 + RSQ7 + RSQ8 + RSQ9}{9}\right) - 1\right] \times 25$$

© Copyright Brigham & Women's Hospital, 2022.
